# Tauroursodeoxycholic acid dampens oncogenic apoptosis induced by endoplasmic reticulum stress during hepatocarcinogen exposure

**DOI:** 10.18632/oncotarget.4377

**Published:** 2015-07-20

**Authors:** Yves-Paul Vandewynckel, Debby Laukens, Lindsey Devisscher, Annelies Paridaens, Eliene Bogaerts, Xavier Verhelst, Anja Van den Bussche, Sarah Raevens, Christophe Van Steenkiste, Marleen Van Troys, Christophe Ampe, Benedicte Descamps, Chris Vanhove, Olivier Govaere, Anja Geerts, Hans Van Vlierberghe

**Affiliations:** ^1^ Department of Hepatology and Gastroenterology, Ghent University, Ghent, Belgium; ^2^ Department of Biochemistry, Ghent University, Ghent, Belgium; ^3^ Infinity Imaging Lab, Ghent University, Ghent, Belgium; ^4^ GROUP-ID Consortium, Ghent University, Ghent, Belgium; ^5^ Translational Cell and Tissue Research, Department of Imaging and Pathology, University of Leuven, Leuven, Belgium

**Keywords:** hepatocellular carcinoma, unfolded protein response, inflammation, chemoprevention, chaperone

## Abstract

Hepatocellular carcinoma (HCC) is characterized by the accumulation of unfolded proteins in the endoplasmic reticulum (ER), which activates the unfolded protein response (UPR). However, the role of ER stress in tumor initiation and progression is controversial. To determine the impact of ER stress, we applied tauroursodeoxycholic acid (TUDCA), a bile acid with chaperone properties. The effects of TUDCA were assessed using a diethylnitrosamine-induced mouse HCC model in preventive and therapeutic settings. Cell metabolic activity, proliferation and invasion were investigated *in vitro*. Tumor progression was assessed in the HepG2 xenograft model. Administration of TUDCA in the preventive setting reduced carcinogen-induced elevation of alanine and aspartate aminotransferase levels, apoptosis of hepatocytes and tumor burden. TUDCA also reduced eukaryotic initiation factor 2α (eIf2α) phosphorylation, C/EBP homologous protein expression and caspase-12 processing. Thus, TUDCA suppresses carcinogen-induced pro-apoptotic UPR. TUDCA alleviated hepatic inflammation by increasing NF-κB inhibitor IκBα. Furthermore, TUDCA altered the invasive phenotype and enhanced metabolic activity but not proliferation in HCC cells. TUDCA administration after tumor development did not alter orthotopic tumor or xenograft growth. Taken together, TUDCA attenuates hepatocarcinogenesis by suppressing carcinogen-induced ER stress-mediated cell death and inflammation without stimulating tumor progression. Therefore, this chemical chaperone could represent a novel chemopreventive agent.

## INTRODUCTION

Hepatocellular carcinoma (HCC) ranks as the second leading cause of cancer-related mortality worldwide and is frequently associated with liver cirrhosis [1, 2]. Resection and transplantation are the only potentially curative treatments available following detection of a small HCC [1]. For the majority of patients with locally advanced disease, however, the multi-kinase inhibitor sorafenib and transarterial embolization are the only approved treatments. Unfortunately, both of these treatments provide a limited survival benefit [1]. Given that risk factors for HCC, such as liver cirrhosis, are fairly well established, chemopreventive strategies may help combat the disease. Ideally, these drugs should be safe for long-term use in the at-risk population. However, no chemopreventive drugs are currently available for HCC [3].

Tauroursodeoxycholic acid (TUDCA) is a hydrophilic bile acid that is produced endogenously in humans at very low levels [4]. TUDCA is synthesized in the conjugation pathway of ursodeoxycholic acid, which is effectively used for treating cholestatic liver diseases, including primary biliary cirrhosis, without major adverse reactions [4]. These hydrophilic bile acids act as bile secretagogues and immunomodulators that can prevent apoptosis induced by several agents, such as hydrophobic bile acids, alcohol, transforming growth factor β_1_, and Fas ligand, in hepatic and non-hepatic cells [5, 6]. In addition, TUDCA protects rat livers during long-term ethanol feeding [7] and human livers from ischemia-reperfusion injury during harvesting and cold storage [8]. The mechanisms involved in the antiapoptotic properties of TUDCA include targeting mitochondrial function and integrity and interactions with the nuclear factor kappa-B (NFκB) signaling pathways [9].

The endoplasmic reticulum (ER) is an important organelle required for cell survival and is highly sensitive to homeostatic alterations. Disruption of ER homeostasis leads to the accumulation of unfolded proteins, which disturb ER function and result in a state known as ER stress [10]. In resting cells, all ER stress receptors are maintained in an inactive state through association with the ER chaperone glucose-regulated protein, 78 kDa (Grp78). Upon ER stress, Grp78 dissociates and triggers the unfolded protein response (UPR), which orchestrates cellular adaptation to stress by inducing transcriptional programs and by repressing global translation by eukaryotic initiation factor 2α (eIf2α) phosphorylation [11]. However, if the stress is too severe, UPR signaling switches from a pro-survival to a pro-apoptotic state, leading to increased pro-apoptotic transcription factor C/EBP homologous protein (Chop) expression and the cleavage of procaspase-12 to its active caspase-12 form [11]. Interestingly, the UPR is activated in several liver diseases, including fatty liver disease, viral hepatitis, alcohol-induced liver injury and HCC [12, 13].

Importantly, TUDCA has been shown to act as a chemical chaperone that decreases UPR signaling and protects hepatocytes against cytotoxicity caused by the ER stress inducer thapsigargin [14, 15]. TUDCA has also been shown to abolish ER stress-induced caspase-12 processing and to subsequently inhibit effector caspases-3/7 activation and apoptosis [15].

Recent studies have demonstrated that hepatocyte apoptosis is a pathogenic event in several liver diseases [13, 16]. Chronically increased hepatocyte apoptosis in genetic mouse models is carcinogenic and leads to compensatory liver regeneration, oxidative stress and DNA hypermethylation [16, 17]. In this study, we investigated the preventive and therapeutic potential of TUDCA in HCC. Our data demonstrate that TUDCA reduces carcinogen-induced liver dysfunction and HCC incidence, likely through the prevention of ER stress-induced apoptosis and inflammation. Importantly, once HCC nodules are established, TUDCA does not modulate tumor growth.

## RESULTS

### Preventive administration of TUDCA reduces HCC burden in an orthotopic mouse model

The ER stress-inducing hepatocarcinogen diethylnitrosamine (DEN) promotes multifocal HCC after 25 weeks of administration [18, 19]. To explore the effects of TUDCA on hepatocarcinogenesis, we supplemented the animals with low or high dose TUDCA in their drinking water. Administration of DEN resulted in reduced body weights and 25% increased mortality (*p* < 0.001; Table 1) compared to saline administration. TUDCA treatment did not affect the weight loss or mortality of DEN-treated mice, and no clinical signs of toxicity were observed in any of the TUDCA-treated groups.

**Table 1:** Mouse body weight (g) (mean ± SD)

Group	Average body weight 25 weeks (g)	Average body weight 30 weeks (g)	Survival (%)
Preventive study			
Saline	31.22 ± 1.39		100
DEN + control	26.75 ± 1.96***		75
DEN + low dose TUDCA	27.39 ± 1.45^#^		83
DEN + high dose TUDCA	24.64 ± 2.17^#^		67
Therapeutic study			
DEN ⇒ control	25.96 ± 3.65	22.92 ± 3.19	67
DEN ⇒ low dose TUDCA	23.86 ± 2.70	22.13 ± 2.57^¥^	75
DEN ⇒ high dose TUDCA	24.53 ± 2.13	23.43 ± 2.61^¥^	67

*** *p* < 0.001: 25 weeks DEN vs. saline.

# not significant compared to DEN + control.

¥ not significant compared to DEN ⇒ control.

DEN, diethylnitrosamine.

**Figure 1:**
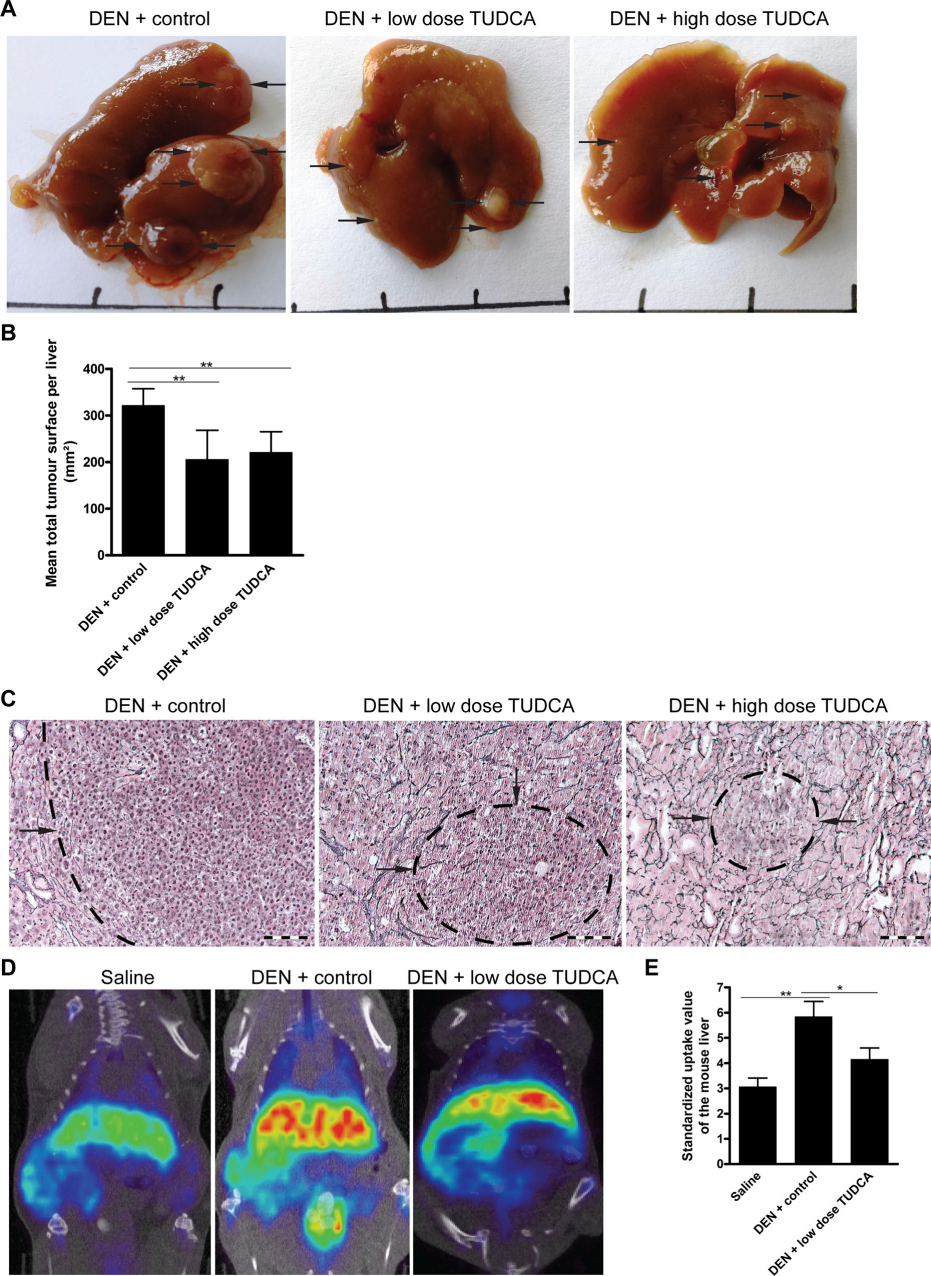
TUDCA prevents the development of HCC during carcinogen exposure **A.** Representative images of livers treated for 25 weeks with the indicated treatments. **B.** Quantitative analysis of the tumor burden as assessed by **C.** Reticulin staining. Scale bar: 100 μm. **D.** ^18^F-Choline positron emission tomography was performed to visualize cell membrane synthesis after the indicated treatments (blue: low, red: high activity). **E.** Quantification of ^18^F-Choline positron emission tomography. Standardized uptake values of the mouse livers are presented as the mean ± SD. One-way ANOVA was applied for statistical analysis. *p < 0.05, **p < 0.01.

DEN-treated mice that received low- or high-dose TUDCA developed fewer nodules per liver (all sizes: 18.9 ± 5.3 after vehicle versus 11.5 ± 3.9 after low-dose TUDCA [*p* < 0.01] and 12.3 ± 4.1 after high-dose TUDCA [*p* < 0.05]). HCC burden, quantified by the loss of reticulin staining, was reduced in DEN-treated mice following the co-administration of low- or high-dose TUDCA (*p* < 0.01; Figure 1A–1C). Choline positron emission tomography, which visualizes cellular membrane biosynthesis, demonstrated that administration of low-dose TUDCA in DEN-treated mice resulted in fewer hepatic loci with high mean standardized uptake values compared with animals receiving vehicle alone (*p* < 0.05; Figure 1D–1E). Repeated DEN administration induces HCC in a background of liver fibrosis [18]. However, no difference in grade of fibrosis, as determined by Sirius Red staining, was found between TUDCA- and vehicle-treated livers (data not shown).

**Figure 2:**
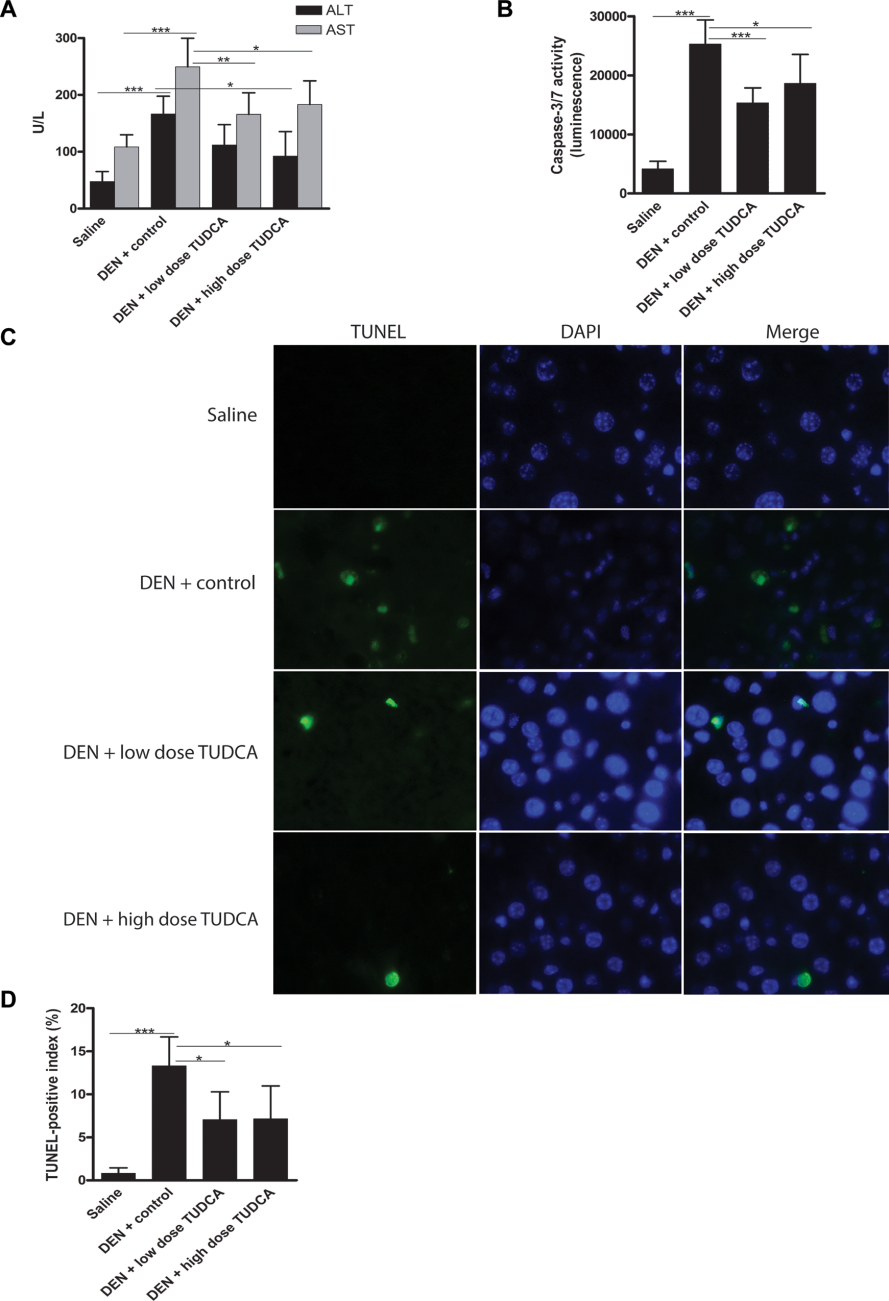
TUDCA reduces DEN-induced apoptosis of hepatocytes **A.** Liver damage was assessed by measuring ALT and AST levels in the serum of mice after the indicated treatments. **B.** Caspase-3/7 activity *ex vivo* (*n* = 8). **C.** TUNEL immunofluorescence and **D.** quantification of the TUNEL-positive index.*p < 0.05, **p < 0.01, ***p < 0.001.

DEN administration increased the levels of serum ALT and AST compared to saline administration (*p* < 0.001; Figure 2A). Importantly, serum ALT and AST levels were reduced in DEN-treated mice receiving TUDCA-supplemented drinking water compared to those receiving regular drinking water (*p* < 0.05; Figure 2A). This suggests that both low- and high-dose TUDCA protect the liver from DEN-induced hepatotoxicity. Collectively, these data indicate that TUDCA decreases the susceptibility of mice to DEN-induced hepatocarcinogenesis in a preventive setting.

### TUDCA attenuates UPR-induced apoptosis in DEN-treated mice

Repeated DEN administration results in significant apoptosis and ER stress in hepatocytes [18, 19]. We measured the activity of effector caspase-3/7 *ex vivo* and, as expected, observed increased levels in DEN-treated mouse livers (*p* < 0.001, Figure 2B). Interestingly, the hepatic caspase-3/7 activity was lower in DEN/TUDCA-treated mice compared to DEN/vehicle-treated mice (Figure 2B). In agreement with these data, TUNEL immunofluorescence demonstrated a significant reduction of TUNEL-positive hepatocytes following TUDCA supplementation (*p* < 0.05, Figure 2C–2D), thus confirming reduced hepatocyte apoptosis upon DEN challenge.

**Figure 3:**
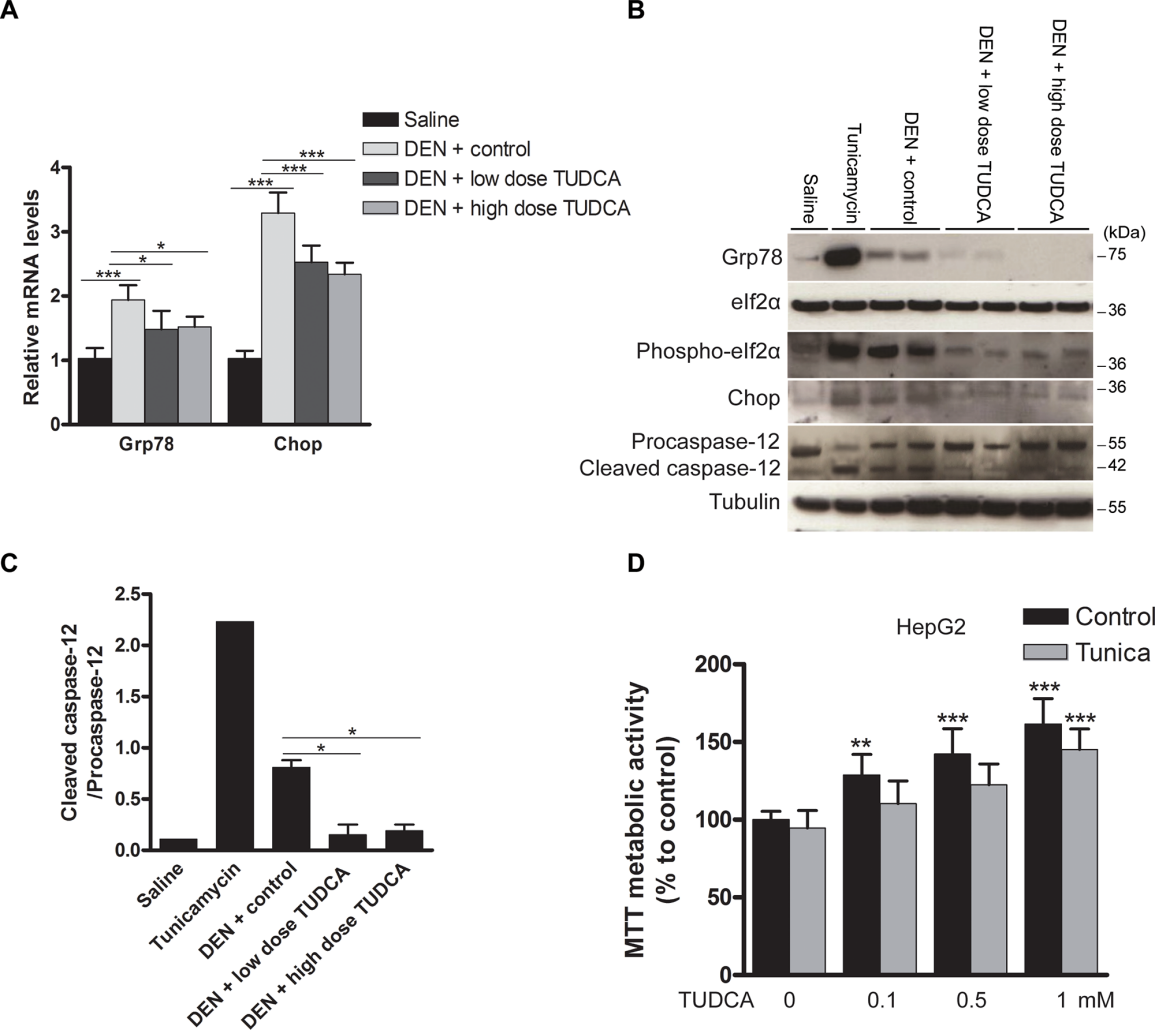
Effect of TUDCA on the hepatic UPR pattern in the DEN-induced mouse model of HCC **A.** Real-time PCR analysis of the UPR targets Grp78 and Chop. **B.** Expression of Grp78, eIf2α, phospho-eIf2α, Chop, procaspase-12 and cleaved caspase-12 was detected using Western blotting. Results are representative of 2 independent experiments. **C.** Cleaved caspase-12/procaspase-12 ratio obtained using densitometric analysis of the Western blot shown in B. **D.** Effect of TUDCA treatment on the MTT metabolic activity of HepG2 cells (treated with tunicamycin (Tunica) or not). *p < 0.05, **p < 0.01, ***p < 0.001. Results are representative of 3 independent experiments.

Grp78 and Chop expression and eIf2α phosphorylation were increased in the mice treated with DEN, reflecting robust UPR activation (Figure 3A–3B). As a positive control, tunicamycin, a nucleoside antibiotic that inhibits protein glycosylation and thereby elicits acute ER stress [11], was administered to naive mice. These control mice showed significantly increased expression of Grp78 and Chop and eIf2α phosphorylation (Figure 3B). Administration of TUDCA during the 25 weeks of carcinogen exposure consistently reduced the expression of Grp78 and Chop and inhibited eIf2α phosphorylation, thereby possibly restoring global translation (Figure 3A–3B). Caspase-12, the central player in ER stress-induced apoptosis [11], was markedly activated by both tunicamycin and DEN administration, whereas TUDCA reduced the DEN-induced cleavage of procaspase-12 (*p* < 0.05; Figure 3C). Overall, these data strongly suggest that TUDCA decreases carcinogen-induced ER stress and thereby attenuates caspase-12-mediated hepatocyte apoptosis.

### TUDCA increases cellular metabolic activity independent of ER stress

To further explore the effect of TUDCA on cell viability and proliferation, HepG2, BWTG3 and Hepa1-6 cells were incubated with a dilution series of TUDCA. As shown by lactate dehydrogenase (LDH) activity in the cell supernatant, TUDCA at 0.1–1 mM did not induce cytotoxicity in HepG2 cells (Supplementary Figure S1A). At 10 mM, however, TUDCA increased the LDH activity (*p* < 0.001). Next, we examined the effect of TUDCA on the MTT metabolic activity of cells under basal conditions. The reduction of tetrazolium salts such as MTT depends on both cellular metabolic activity and proliferation rate. Intriguingly, 0.1–1 mM TUDCA increased the MTT metabolic activity of HepG2 cells in a dose-dependent manner to supranormal levels of up to 150% (*p* < 0.001; Figure 3D). Incorporation of the thymidine analogue bromodeoxyuridine (BrdU) into the DNA of HepG2 cells under basal conditions showed that TUDCA modestly decreased the cell proliferation rate at 1 mM (*p* < 0.05, Supplementary Figure S1B). Furthermore, we directly counted HepG2 cells after 48 h of incubation with 0.1–1 mM TUDCA and found no difference in cell number compared to controls (data not shown). The MTT metabolic activity and BrdU incorporation experiments were repeated in BWTG3 and Hepa1-6 cells with similar results (Supplementary Figure S1C–S1F). These data suggest that, under basal conditions, TUDCA dose-dependently enhances cellular metabolic activity without increasing the absolute number of cells.

Next, we examined whether TUDCA would have the same effects in the presence of ER stress [11]. Tunicamycin (0.5 μg/ml) did not alter MTT metabolic activity or LDH release in HepG2 cells, suggesting no inherent cytotoxicity at the concentration used (Figure 3D and Supplementary Figure S1A, respectively). However, tunicamycin did impair BrdU incorporation (*p* < 0.01; Supplementary Figure S1B), confirming the well-known ER stress-mediated induction of cell cycle arrest [11]. Finally, TUDCA was able to enhance cellular metabolic activity even in the presence of acute ER stress (Figure 3D and Supplementary Figure S1C–S1D); however, this had no effect on the antiproliferative effect of ER stress (Supplementary Figure S1B and Supplementary Figure S1E–S1F).

### TUDCA does not reduce oxidative stress-induced cell death or DEN-induced oxidative stress

TUDCA has been shown to exert cytoprotective effects in different models by reducing oxidative stress [20], and ER stress is closely connected to the oxidative stress response [10]. Thus, we evaluated the effect of TUDCA on the cytotoxicity of H_2_O_2_-induced oxidative stress in HepG2 cells. Cell viability declined following the addition of 1–5 mM of H_2_O_2_ in a dose-dependent manner (Supplementary Figure S2A). In contrast to the established antioxidant properties of N-acetylcysteine, co-incubation with 0.1 or 1 mM TUDCA did not protect HepG2 cells against H_2_O_2_-induced cell death (*p* < 0.01; Supplementary Figure S2A).

In the orthotopic HCC model, TUDCA supplementation did not alter the expression levels of DEN-induced antioxidant response genes such as glutathione-S-transferase A1 (*Gsta1*), glutathione-S-transferase A2 (*Gsta2*), nuclear factor erythroid-derived 2 like 2 (*Nfe2l2*) and glutamate-cysteine ligase (*Gclc*; Supplementary Figure S2B). Malondialdehyde (MDA) is a toxic product of reactions between reactive oxygen species (ROS) and polyunsaturated lipids that is commonly used as a biomarker to quantify oxidative stress. DEN administration resulted in substantial accumulation of MDA protein adducts (*p* < 0.001), and this was found to be unaltered by TUDCA supplementation (Supplementary Figure S2C). Thus, the chemopreventive action of TUDCA seems to be independent of oxidative stress.

### TUDCA does not affect DEN-induced autophagic flux

ER stress is able to induce autophagy [21], which has been shown to protect against hepatocarcinogenesis [22]. We therefore tested whether TUDCA modulates autophagy *in vivo* (Supplementary Figure S3). DEN-treated liver tissue exhibited enhanced expression of Beclin-1 and increased conversion of LC3B-I to LC3B-II, indicating the activation of autophagic signals. The cellular content of p62, a receptor and substrate of selective autophagy, is a critical indicator of autophagic flux [23]. Immunoblotting for p62 showed that DEN slightly decreased the hepatic p62 content (Supplementary Figure S3A). TUDCA administration did not alter the stably elevated LC3 conversion and reduced p62 content. Thus, TUDCA was unable to alter the enhanced autophagic flux.

### TUDCA attenuates DEN-induced hepatic inflammation

**Figure 4:**
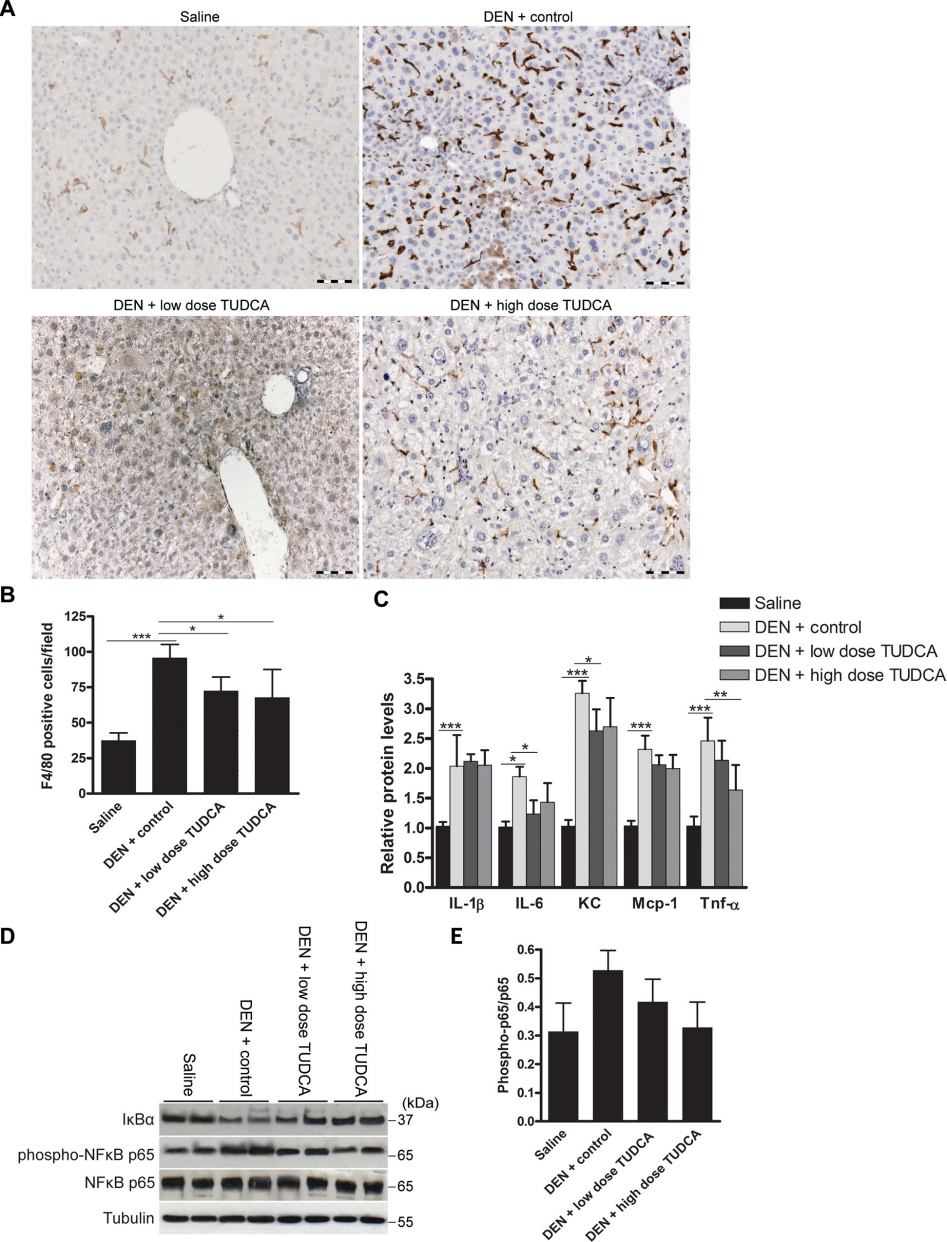
Effect of TUDCA on DEN-induced hepatic inflammation **A.** Immunostaining for F4/80 and **B.** quantification of F4/80-positive macrophages in the liver after the indicated treatments (*n* = 5). **C.** Determination of the indicated hepatic cytokine levels by multiplex microbead immunoassay (*n* = 8). Values represent the mean ± SD. *p < 0.05, **p < 0.01, ***p < 0.001. **D.** Expression of IκBα, phospho-NFκB p65 and NFκB p65 was detected using Western blotting. Results are representative of 2 independent experiments. **E.** Phospho-NFκB p65/ NFκB p65 ratio obtained using densitometric analysis of the Western blot shown in D.

Human HCC usually develops in the background of chronic hepatic inflammation [1]. Repeated DEN-induced murine tumors share similar pathogenesis [18], where pro-inflammatory cytokines, such as interleukin-6 (IL-6) and tumor necrosis factor-α (TNF-α), promote tumor development [24]. Indeed, accumulating evidence suggests extensive cross-talk between the UPR and the inflammatory response [25]. Chop-deficient livers have been shown to exhibit reduced inflammation and hepatocarcinogenesis [26]. Because TUDCA abolished the DEN-induced Chop expression in our study, we hypothesized that TUDCA might alter DEN-induced hepatic inflammation. Immunohistochemical staining demonstrated robust accumulation of F4/80+ macrophages in the livers of animals treated with DEN for 25 weeks (Figure 4A–4B). However, TUDCA significantly decreased the number of liver-infiltrating macrophages (*p* < 0.05). Using multiplex microbead immunoassays, we observed that hepatic expression of inflammatory cytokines, including interleukin-1 beta (IL-1β), IL-6, keratinocyte-derived chemokine (KC), monocyte chemoattractant protein-1 (Mcp-1), and TNF-α, was augmented following DEN administration. Importantly, IL-6 (*p* < 0.05), KC (*p* < 0.05) and TNF-α (*p* < 0.01) levels were reduced following TUDCA supplementation (Figure 4C).

Elevated eIf2α phosphorylation leads to translational repression of the NF-κB inhibitor IκBα and thereby promotes NF-κB signaling [27], which exerts a pro-carcinogenic role in inflammation-related hepatocarcinogenesis [28]. Using salubrinal, which inhibits eIf2α de-phosphorylation, we confirmed that sustained eIf2α phosphorylation leads to translational repression of IκBα in HepG2 cells (data not shown). Because DEN-induced eIf2α phosphorylation was abolished by TUDCA supplementation (Figure 3B), we assessed the effect of TUDCA on hepatic IκBα expression and NF-κB activation to investigate this potential mechanism of action. Interestingly, TUDCA supplementation restored IκBα expression and slightly decreased phospho-NFκB p65 levels, the active form of NF-κB, in the DEN-treated livers (Figure 4D–4E). These data indicate that TUDCA suppresses the immune response to DEN-induced liver injury by reducing phospho-eIf2α-mediated repression of IκBα translation.

### TUDCA alters invasiveness *in vitro*

**Figure 5:**
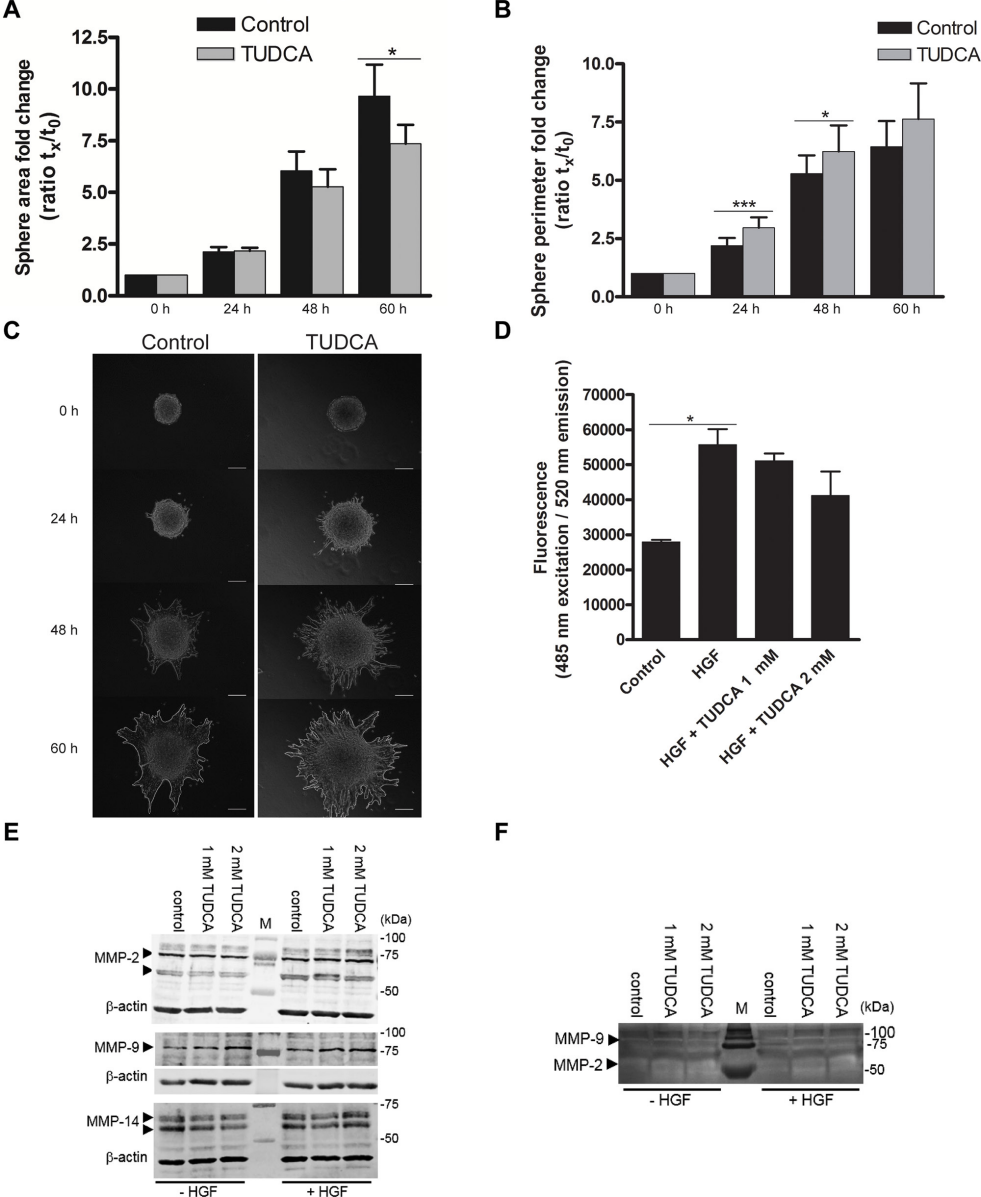
Effects of TUDCA on HCC cell invasion **A.** The area and **B.** length of the outside boundary (perimeter) of collagen-embedded Hepa1-6 spheroids (*n* = 9) was measured at 0, 24, 48 and 60 h of incubation with 2 mM TUDCA or control medium. Results are representative of 2 independent experiments. **C.** Representative images of invasive capacity of cells present in a collagen-embedded multicellular spheroid are shown at different time points. The multicellular spheroids are approx. 150 μm in diameter at start. At 60 h, the spheroid perimeter is marked. Scale bar is 50 μm. **D.** Boyden chamber invasion assay with Hepa1-6 cells following 48 h of incubation with control medium, control medium with chemoattractant hepatocyte growth factor (HGF) without or with 1–2 mM TUDCA. Samples were run in quadruplicate. **E.** Western blot analysis of MMP-2, -9 and -14 levels in Hepa1-6 cell lysates of control cells and cells treated with 1 or 2 mM TUDCA (48 h), in the presence or absence of 50 ng/ml HGF stimulation. Arrows indicate MMP-2 (72 and 63–66 kDa), MMP-9 (78–82 kDa) and MMP-14 (66–57 kDa). The β-actin signal is used as loading control. **F.** Gelatin zymography of concentrated culture medium of Hepa1-6 control cells or cells treated with 1 or 2 mM TUDCA (48 h), in the presence or absence of 50 ng/ml HGF stimulation. The white bands indicate MMP-activity; arrowheads: signal for MMP-9 (glycosylated, 92 kDa) and MMP-2 (58/62 kDa, active). Values represent the mean ± SD. *p < 0.05, ***p < 0.001.

Because ER stress was previously linked to cell invasion [29, 30], we questioned whether TUDCA could modify HCC cell invasion *in vitro*. Therefore, we examined the effect of TUDCA in a hepatocyte growth factor-induced invasion assay in which spheroids of the Hepa1-6 HCC cell line embedded in collagen matrix were observed over time (Figure 5A–5C). TUDCA decreased the sphere area after 60 h of incubation (*p* < 0.05; Figure 5A), whereas the perimeter of the TUDCA-treated spheres increased compared with controls after 24 (*p* < 0.001) and 48 h (*p* < 0.05) of incubation (Figure 5B). Although TUDCA may have decreased sphere area by affecting cell proliferation in this context (e.g., as observed for HepG2 in Figure 3B), the larger perimeter combined with a smaller area suggests an altered invasive phenotype. This result was also supported by the more irregular sphere shape observed upon invasion in the presence of TUDCA (Figure 5C). However, in a Boyden chamber assay, addition of 1–2 mM of TUDCA induced no significant alterations in the hepatocyte growth factor-stimulated invasion of the HCC cells over a 48-hour period (Figure 5D).

Degradation of the extracellular matrix is one pivotal step and occurs due to the actions of matrix metalloproteinases (MMP). MMP-2, MMP-9 and MMP-14 enzymes play important roles in the degradation of the extracellular matrix and exist extensively in HCC tissues. MMP profiling showed that addition of TUDCA did not alter the protein levels of MMP-2, -9 and -14 (Figure 5E). Accordingly, we observed no changes in the extracellular MMP-2 and -9 activity in the concentrated conditioned medium (Figure 5F). In conclusion, although TUDCA alters the invasive phenotype, TUDCA does not induce increased HCC cell invasion.

### TUDCA did not affect tumor progression

**Figure 6:**
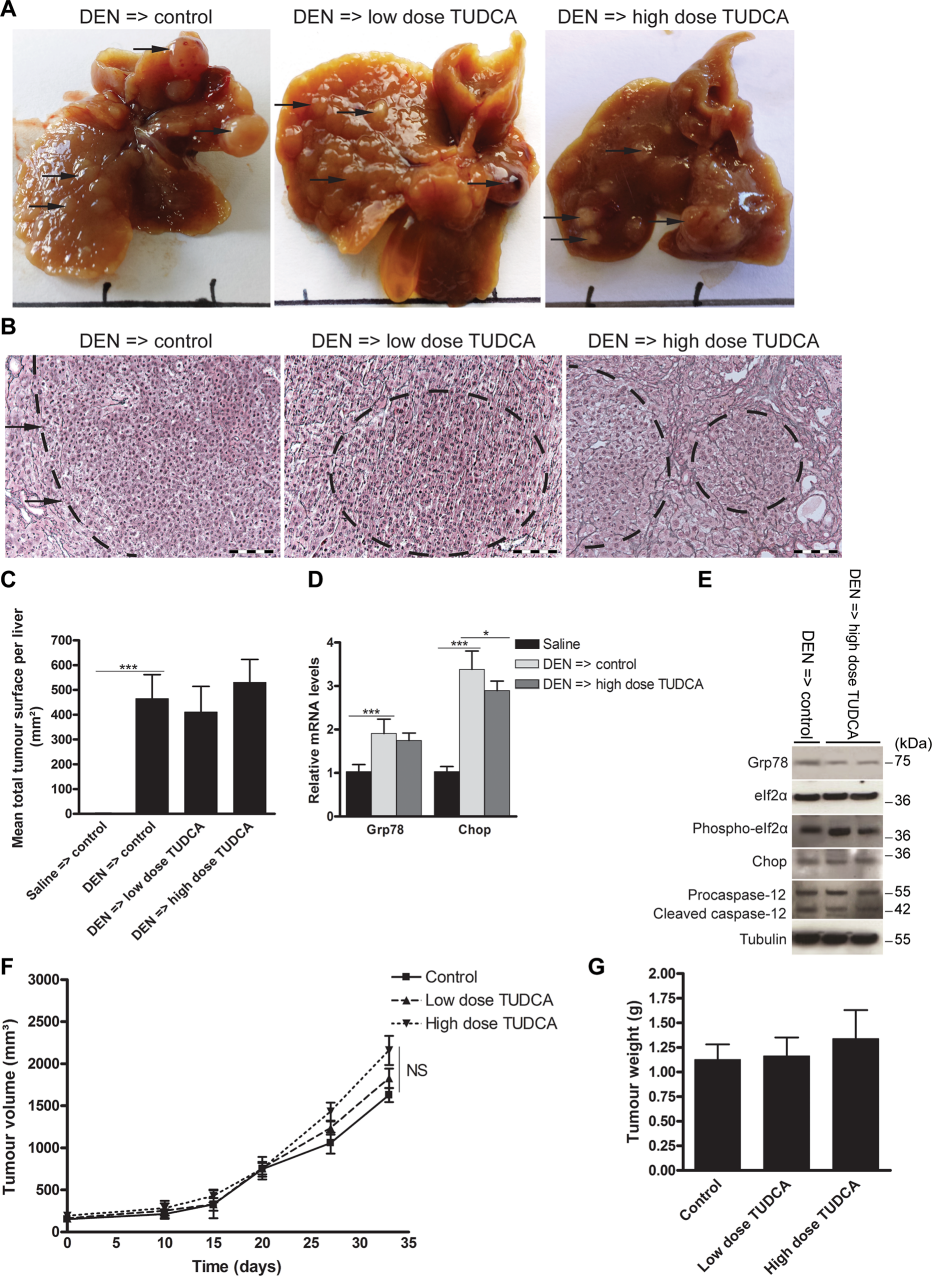
Effect of TUDCA on the orthotopic and xenograft mouse models of HCC progression **A.** Representative images of murine livers treated for 25 weeks with DEN injections followed by 5 weeks of TUDCA-supplemented or control drinking water. **B.** Reticulin staining of the DEN-treated livers for **C.** quantification of tumor burden. Scale bar: 100 μm. **D.** Real-time PCR analysis of the UPR targets Grp78 and Chop. **E.** Expression of Grp78, eIf2α, phospho-eIf2α, Chop, procaspase-12 and cleaved caspase-12 was detected using Western blotting. Results are representative of 2 independent experiments. **F.** Effect of indicated treatments on growth of HepG2 xenografts in athymic nude mice (*n* = 6). The volume of each tumor was measured for 33 days. Values represent the mean ± SD. **G.** At the end of the treatment period, animals were sacrificed and tumor weights were recorded. *p < 0.05, **p < 0.01.

Given our findings that TUDCA attenuated UPR-induced apoptosis and increased cellular metabolic activity, we evaluated whether TUDCA could stimulate tumor progression via its cytoprotective effect. To assess the effect of TUDCA on established tumors, we used orthotopic and xenograft mouse models of HCC. Following orthotopic HCC induction with 25 weeks of DEN, 5 weeks of low- or high-dose TUDCA supplementation had no significant effect on mortality (Table 1) or tumor number (all sizes: 17.4 ± 4.9 after vehicle versus 19.7 ± 4.5 after low-dose TUDCA and 18.8 ± 5.3 after high-dose TUDCA). Accordingly, microscopic quantification confirmed that TUDCA produced no significant effects on HCC burden (Figure 6A–6C). Next, we investigated the effects of 5 weeks of TUDCA supplementation following 25 weeks of DEN-induced tumor development on the UPR (Figure 6D–6E). TUDCA did not alter the protein levels of Chop or phospho-eIf2α in HCC-bearing livers (Figure 6E). Moreover, no difference in the apoptosis rate was observed (data not shown). These results suggest that TUDCA was unable to restore ER function after prolonged hepatocarcinogen exposure.

In the HepG2 xenograft model, TUDCA supplementation for 5 weeks did not significantly modify tumor growth compared to control treatment (Figure 6F–6G), and no mortality occurred in any of these groups. Finally, no metastases were detected in any group of the preventive or therapeutic settings of the orthotopic or of the HepG2 xenograft model.

## DISCUSSION

**Figure 7:**
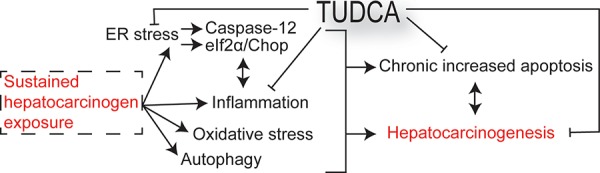
Schematic overview outlining the mechanisms of the chemopreventive effects of TUDCA

There is an urgent need for innovative preventive and therapeutic options for HCC [3]. Previous studies have demonstrated that the chemical chaperone TUDCA serves as a cytoprotective agent by reducing ER stress and apoptosis [5, 7, 8, 14]. In this study, we evaluated the preventive and therapeutic potential of TUDCA and its effect on carcinogen-induced ER stress in HCC. Our results reveal that TUDCA supplementation during carcinogen exposure reduces the carcinogen-induced apoptosis of hepatocytes and HCC incidence (Figure 7). Furthermore, it does not stimulate the progression and invasion of established tumors

A recent double-blind randomized trial demonstrated that daily TUDCA therapy (750 mg) for 6 months is safe and appears to be an effective liver cirrhosis treatment; in particular, it improved several biochemical parameters [31]. In addition, TUDCA therapy in cirrhotic patients awaiting liver transplantation supported their functional stability during the wait time [32]. Our results suggest that TUDCA therapy in patients with a high HCC risk, such as cirrhotic patients with continuous exposure to carcinogens, not only improved liver function but could also prevent HCC incidence.

We selected a low dosage (60 mg/kg/day), which corresponds to dosages administrable and tolerable for humans, and a high dosage (300 mg/kg/day) to assess dose-dependent effects and potential side effects. Both low- and high-dose TUDCA administration decreased tumor burden in our mouse models. However, the trend of reduced weight and survival was only observed in the high-dose TUDCA group. This reduction in body weight was also observed in a mouse model of retinitis pigmentosa, where animals were treated with high-dose TUDCA (500 mg/kg/day) [33]. Additionally, TUDCA has been shown to increase energy expenditure by promoting intracellular thyroid hormone activation [34]. Therefore, a low dose of TUDCA is advisable for chemoprevention.

Chemically improving the ER folding capacity by TUDCA administration was previously shown to protect from UPR signaling and ER stress-induced apoptosis, which are primarily regulated by the phospho-eIf2α\Chop cascade [10, 15]. Although Chop plays critical pro-apoptotic roles, it was recently shown to promote carcinogenesis in a DEN-induced model of HCC [26]. Accordingly, TUDCA efficiently diminished Chop expression and tumorigenesis during carcinogen exposure in our study.

In hepatocytes, DEN is metabolized by cytochrome P450 2E1 through a ROS-generating reaction that induces liver injury and DNA damage [35, 36]. Subsequently, danger signals released from injured hepatocytes induce liver inflammation. Accordingly, certain antioxidants, such as N-acetylcysteine [35] or lycopene [37], have been shown to attenuate DEN-induced hepatocarcinogenesis. Of note, N-acetylcysteine was recently shown to accelerate lung cancer progression in mice [38]. Because TUDCA did not affect oxidative stress-induced cytotoxicity *in vitro* or hepatic oxidative stress *in vivo*, we presume that the chemopreventive effect of TUDCA was not mediated by directly antagonizing oxidative stress but rather by modulating ER stress-induced apoptosis. Therefore, the combination of TUDCA with antioxidants could represent a dual-targeting chemopreventive strategy for HCC.

HCC has been characterized as a chronic inflammation-driven cancer, and chemically induced models have revealed the crucial roles of inflammatory signaling in disease onset and severity [35]. Therefore, we assessed whether TUDCA affected tumor-immune system crosstalk. TUDCA administration resulted in a decrease in hepatic macrophage infiltration and IL-6, KC and TNF-α levels. These results suggest that TUDCA attenuates inflammation in response to DEN-induced liver injury and thus interrupts positive feedback from inflammation-induced ER stress and UPR-induced inflammation [13]. Interestingly, a similar effect of TUDCA on macrophage infiltration and TNF-α expression was also recently observed in a model of ER stress-mediated steatohepatitis-induced HCC [39]. However, additional studies are needed to uncover the precise mechanism of the interaction between ER stress and inflammation and the effects of TUDCA on these signaling pathways.

Uncontrolled regeneration of hepatocytes, which occurs after repeated cycles of cell death and compensatory proliferation in chronic hepatitis, appears to be an important factor in hepatocarcinogenesis [16, 40]. Apparently, increased hepatocyte apoptosis contributed to the development of HCC in our DEN-induced mouse model. TUDCA interrupts the positive feedback from UPR-mediated apoptosis-induced hepatocarcinogenesis, leading to UPR activation by tumor microenvironmental stresses [11].

Finally, an oral treatment option for preventing HCC would be highly beneficial for cirrhotic patients with high HCC risk. In this study, we showed that TUDCA could represent a clinically applicable chemopreventive agent for HCC. Whether hepatocyte susceptibility to other carcinogens, such as viral replication or alcohol, can be limited by chemical chaperones will be of great interest.

In conclusion, our study demonstrates that supplementation with TUDCA diminishes carcinogen-induced hepatotoxicity and prevents tumor induction by, at least partially, alleviating positive feedback from ER stress, inflammation and apoptosis in carcinogen-injured liver tissue.

## MATERIALS AND METHODS

### Animal studies

#### Ethics statement

The investigation was conducted in accordance with the ethical standards and according to the Declaration of Helsinki as well as the national and international guidelines. The investigation was also approved by the institutional review board of Ghent University (ECD 11/52).

#### Orthotopic model

Wild-type 129S2/SvPasCrl mice were purchased from Charles River (Belgium) and maintained as previously described [18]. In the preventive arm of the study, 5-week-old male mice were randomly divided into 4 groups (*n* = 12 in each group). Three groups received weekly intraperitoneal DEN (35 mg/kg, in saline) injections for 25 weeks with either regular drinking water or drinking water supplemented with varying amounts of TUDCA (low dose of 60 mg/kg/day or high dose of 300 mg/kg/day). Mice in the control group received 25 weeks of saline injections and regular drinking water. In the therapeutic arm of the study, mice received DEN for 25 weeks before being treated with saline- or TUDCA-supplemented (low or high dose) drinking water for 5 weeks (*n* = 12 in each group). In addition, 30 μg of tunicamycin was intraperitoneally injected in four naive 30-week-old male 129S2/SvPasCrl mice 72 h before sacrifice. Blood was collected from the retro-orbital sinus under isoflurane anesthesia. After macroscopic evaluation and quantification of hepatic tumor number, all organs were fixed in 4% phosphate-buffered formaldehyde (Klinipath) and embedded in paraffin or snap frozen in liquid nitrogen. Hematoxylin/eosin and reticulin staining were performed to assess tumor burden, and the results were evaluated by two independent observers. Sirius Red staining enabled fibrosis assessment according to Metavir scoring. Serum alanine and aspartate aminotransferase (ALT and AST, respectively) levels were measured at the Lab of Clinical Biology, Ghent University Hospital.

### Xenograft model

HepG2 cells (6×10^6^) were re-suspended in 100 μl of serum-free media and mixed with 100 μl of Matrigel (BD Biosciences, Bedford, MA, USA). The cell preparation was injected subcutaneously into the right flank of 8-week-old male athymic nude mice. Tumor volumes were calculated using the following formula: volume (mm^3^) = ab^2^/2; where b is the smaller dimension. When the mean tumor volume reached 150 mm^3^, animals were randomized into three groups (*n* = 6 in each group) as follows: regular drinking water, low-dose (60 mg/kg/day) TUDCA-supplemented drinking water and high-dose (300 mg/kg/day) TUDCA-supplemented drinking water. Tumor dimensions were recorded two times per week with a digital caliper starting on the first day of treatment. Tumor weights were recorded at the time of sacrifice.

### Cell culture

HepG2, BWTG3 and Hepa1-6 (ATCC, Virginia, USA) cells were cultured in DMEM supplemented with 10% fetal bovine serum (Life Technologies, Ghent, Belgium). Cells were incubated for 48 h with tunicamycin (0.5 μg/ml), TUDCA (0.1–10 mM, Calbiochem, MA, USA), salubrinal (50 μM, Tocris, Bristol, UK), N-acetylcysteine (5 mM), H_2_O_2_ (1–5 mM) or equal volumes of solvent. For direct cell counting, cells were trypsinised and counted in trypan blue. All reagents were obtained from Sigma (Diegem, Belgium) unless stated otherwise. Experiments were performed in quadruplicate and independently repeated three times.

Detailed information regarding choline positron emission tomography, caspase-3/7 activity, TUNEL apoptosis, RNA extraction, quantitative real-time PCR, western blot analysis, LDH, MTT, BrdU incorporation, lipid peroxidation, immunohistochemistry, multiplex microbead and spheroid invasion assays is provided in the Supporting Information.

### Statistics

Statistical analyses were performed using SPSS version 21 (SPSS, Chicago, USA). Data are presented as the mean ± SD or fold change relative to expression in controls. Normally distributed data were subjected to unpaired student’s *t*-tests. Multiple groups were compared by one-way analysis of variance (ANOVA) with Bonferroni correction. Non-normally distributed data were tested using the Mann-Whitney U test. The chi-squared test was used to compare mortality. Student’s paired *t*-test was used to compare area or perimeter fold change. Reported *p*-values were two-sided and considered significant when less than 0.05.

## SUPPLEMENTARY MATERIALS AND METHODS



## List of abbreviations

HCC: hepatocellular carcinoma
TUDCA: tauroursodeoxycholic acid
NFκB: nuclear factor kappa-B
ER: endoplasmic reticulum
UPR: unfolded protein response
Grp78: glucose-regulated protein, 78 kDa
eIf2α: eukaryotic initiation factor 2α
Chop: C/EBP homologous protein
DEN: diethylnitrosamine
LDH: lactate dehydrogenase
BrdU: bromodeoxyuridine
Gsta1: glutathione-S-transferase A1
Gsta2: glutathione-S-transferase A2
Nfe2l2: nuclear factor erythroid-derived 2, like 2
Gclc: glutamate-cysteine ligase
