# Supplementary material for: Tauroursodeoxycholic acid dampens oncogenic apoptosis induced by endoplasmic reticulum stress during hepatocarcinogen exposure
Source: Oncotarget. 2015 Jul 20;6(29):28011–25. doi: 10.18632/oncotarget.4377 (PMC4695041; doi:10.18632/oncotarget.4377)
Supplement: Supplementary file 1 [file oncotarget-06-28011-s001.pdf]

SUPPLEMENTARY MATERIALS AND METHODS

Choline positron emission tomography

In vivo tests were performed using positron emission tomography (PET). PET-CT acquisitions were performed using a triple-modality Triumph II micro-PET/SPECT/CT scanner (Gamma Medica-Ideas). This state-of-the-art scanner consists of a micro-PET module (LabPET8) with 2×2×10-mm LYSO/LGSO scintillators in an 8-pixel, quad-APD detector module arrangement. This system can deliver a 1.4-mm spatial resolution in rodents at a sensitivity of 4%, thereby covering a field-of-view of 10 cm transaxially by 8 cm axially. The micro-CT portion consists of a high-resolution micro-CT tube with a focal spot size variable between 20 and 129 micrometer combined with a flat-panel CsI detector.

Animals (3 groups with $n = 3$) were injected in the tail vein with 516 ± 25 µCi of [18F]-fluoromethylcholine ([18F] FMCH) (Laboratory of Radiopharmacy, Ghent University, Belgium) immediately prior to micro-PET scanning at the beginning of a 30-minute dynamic acquisition. For anatomical localization, a micro-CT scan was sequentially acquired using 256 projections over 360 degrees at 75 kVp/240 microA and 1.3× magnification with a focal spot size of 50 micrometer. The resulting PET data were reconstructed using 30 iterations of the Maximum-Likelihood Expectation-Maximization algorithm in a 160 × 160 × 63 matrix with a 0.5 × 0.5 × 1.175-mm voxel size. No additional spatial filtering was applied. The acquired CT projection images were reconstructed using a filtered back-projection algorithm. All images were fused and analyzed using AMIDE software.

Caspase-3/7 activity assay

The enzymatic activation of effector caspase-3/7 was evaluated in fresh liver tissue using the Caspase-Glo® 3/7 assay Kit (Promega, Leiden, The Netherlands) following the manufacturer's instructions. Experiments were performed in sextuplicate.

TUNEL apoptosis assay

Embedded liver sections were deparaffinized, rehydrated through graded alcohol, and permeabilized with 0.1% TritonX-100 at room temperature (8 min incubation). Slides were rinsed twice in a phosphate-buffered saline (PBS). Apoptosis was detected through in situ terminal deoxynucleotidyl transferase (TdT)-mediated deoxyuridine triphosphate (dUTP) nick end-labeling (TUNEL), using the In Situ Cell Death Detection Kit (Roche, Vilvoorde, Belgium). Slides were incubated with the TUNEL reaction mixture containing TdT and fluorescein-dUTP for 1 h at 37°C; and subsequently rinsed three times in PBS. The sections were mounted with an antifade solution containing 4′, 6-diamidino-2-phenylindole (DAPI) (Vectashield, Lab Consult) for nuclear staining. Images were acquired on a Nikon TE300 inverted epifluorescence microscope with a x20 objective and equipped with a Nikon DS-Ri1 cooled color CCD camera (Nikon Belux, Brussel, Belgium). For quantification of apoptotic cells, five random areas from each slide and 5 slides per liver were analyzed. Cells containing green fluorescence and either nuclear condensation or chromatin fragmentation (without nuclear morphological changes) were identified as apoptotic cells. Results were expressed as TUNEL-positive index (number of TUNEL-positive cells per number of total cells quantified from DAPI-positive counts).

Total RNA extraction

Total RNA was extracted from all samples using the RNeasy Mini Kit (Qiagen, Westburg BV, The Netherlands) with on-column DNAse treatment (Qiagen). Needle homogenization was performed. The purity and quantity of total RNA was assessed using spectrophotometry (Nanodrop; Thermo Scientific, Wilmington, USA). The ratio of absorbance at 260 and 280 nm was used to define RNA purity; samples with a 260:280 ratio between 1.8 and 2.0 were accepted.

Quantitative real-time PCR

One microgram of total RNA was converted to single strand cDNA by reverse transcription (iScript, BioRad, California, USA) with oligo (dT) and random priming. The cDNA was diluted 1/10 and used for real-time quantification using SYBR Green (Sensimix, Bioline Reagents Ltd, London, UK) and 250 nM of each primer. A two-step program was run on a LightCyclerR 480 (Roche). Cycling conditions were 95°C for 10 minutes and 45 cycles of 95°C for 10 seconds followed by 60°C for 1 minute. Melting curve analysis confirmed primer specificities. All reactions were performed in duplicate. Fold-change expression was calculated using the ΔΔCT method instructed by Applied Biosystems. First, the normalization of the threshold cycle (CT) values of the target gene was performed with the CT of GAPDH in the same samples (ΔCT = CT target – CT GAPDH). The expression was normalized again with the control (ΔΔCT=ΔCT – ΔCT control), and the fold change was calculated ($2^{-\Delta\Delta CT}$). The PCR-efficiency of each primer pair was calculated using a standard curve of reference cDNA. Amplification efficiency was determined using the formula $10^{-1/slope}$. The primer set sequences are listed in Supplementary Table S1.

Western blotting analysis

Total protein extract was obtained by dissolving cells in RIPA buffer (1x PBS, 1% NP-40, 0.5% sodium deoxycholate, 0.1% SDS and 1× complete protease inhibitors (Roche Diagnostics, Mannheim, Germany)). The total protein yield was determined using Bradford reagent (BioRad, California, USA). Approximately 25–50 µg of protein was loaded and separated by SDS-PAGE. The proteins were transferred to a PVDF membrane (Millipore), which was subsequently blocked and incubated with specific antibodies (Supplementary Table S2) in 5% non-fat milk followed by horseradish peroxidase-conjugated secondary antibodies. UPR-related antibodies were validated using tunicamycin. ECL detection reagent (Amersham Life Science, New Jersey, USA) was used to visualize the specific proteins. The densitometric analysis was performed using ImageJ [1].

Lactate dehydrogenase and MTT assays

HepG2, Hepa1-6 and BWTG3 cells were seeded in 96-well plates at $5x10^4$ cells/well in Dulbecco's Modified Eagle's medium supplemented with 10% fetal bovine serum and incubated the next day with the indicated treatments. After 48 h, 100 µl of supernatant was collected for lactate dehydrogenase (LDH) measurement according to the manufacturer's protocol (Biovision, California, USA). LDH is released into the culture medium following the loss of membrane integrity resulting from either apoptosis or necrosis.

Cell metabolic activity was determined using the MTT assay (Roche Diagnostics, Mannheim, Germany). After supernatant collection, the reagent was added, and the cells were incubated for 4 h at 37°C. The absorbance of the DMSO-solved MTT crystals was measured at 450 nm against a background control at 655 nm (Multiskan Ascent, Leuven, Belgium). Experiments were performed in sextuplicate.

Bromodeoxyuridine incorporation assay

Proliferative activity was assessed by 5-bromo-2-deoxyuridine (BrdU) labeling (Roche, Mannheim, Germany) following the manufacturer's instructions. The absorbance obtained in the ELISA was measured at 450 nm against a background control at 690 nm (Multiskan Ascent, Leuven, Belgium). Experiments were performed in sextuplicate.

Hepatic lipid peroxidation

Lipid peroxidation in liver was monitored by quantifying thiobarbituric acid reactive substances (TBARS). About 25 mg of liver was homogenized in 2-fold volumes of ice-cold PBS, and TBARS were determined via the OXltek TBARS kit (ZeptoMetrix, Buffalo, NY). Sample homogenates, as well as malondialdehyde (MDA) standards, were incubated with sodium dodecyl sulfate (SDS) solution and thiobarbituric acid at 95°C for 60 min and then chilled on ice for 10 min. Then, samples were centrifuged at 1800 g for 15 min and the supernatants were transferred to a 96-well plate and quantified at 532 nm. MDA equivalents were expressed as nmol of MDA equivalents per gram liver.

Immunohistochemistry

A portion of the unprocessed liver tissues were fixed in 4% paraformaldehyde, dehydrated, embedded in paraffin, and sliced into 4 µm thick sections. After dewaxing in xylene and rehydrating in a graded alcohol series, the sections were processed for antigen retrieval by heating in citrate buffer, pH 6.0, for 30 min at 95°C. Immunodetection of F4/80 was carried out in a humidified chamber, as follows ($n = 5$ per group). After three washes in PBS for 5 min each, the sections were covered with 3% bovine serum albumin (BSA) and incubated at 37°C for 30 min. Then, primary antibody replaced the BSA and the slides were incubated at 4°C overnight. After three washes in PBS for 5 min each, the sections were incubated with goat anti-rabbit IgG labeled with horseradish peroxidase-streptavidin complex (Invitrogen, CA, USA) at 37°C for 30 min. After three washes in PBS for 5 min each, the immunoreactive proteins were detected by staining with DAB (Abcam, Cambridge, UK) according to the manufacturer's instructions. Ten magnifications (200×) fields per liver were assessed for the determination of the average number of F4/80-positive cells/field per liver.

Cytokine detection by multiplex microbead immunoassay

A magnetic bead-based multiplex immunoassays (Bio-Plex) (Bio-Rad Laboratories, California, USA) was used for cytokine measurement in murine whole liver lysates following manufactures' instructions ($n = 8$ per group). Cytokines measured were: IL-1β, IL-6, KC, monocyte chemoattractive protein (Mcp-1/CCL2), and Tnf-α. Concentrated mouse recombinant cytokines were provided by the vendor (Bio-Rad Laboratories) and was used to establish standard curves. Cytokine levels were determined using a multiplex array reader from Luminex™ Instrumentation System (Bio-Plex® MAGPIX™ Multiplex Reader from Bio-Rad Laboratories). The analyte concentration was calculated using software provided by the manufacturer (Bio-Plex Manager Software).

Spheroid invasion assay

Multicellular spheres of Hepa1-6 cells were generated in agarose gels with microwells (330 microwells/gel, 800 µm diameter/well) that were made using polydimethyl-siloxane (PDMS) micro-molds (MicroTissues Inc, Providence, Rhode Island, USA) as described [2]. In brief, 2.75 ml of 2% (w/v) agarose solution was pipetted into each PDMS micro-mold. The resulting agarose gel was separated from the PDMS micro-mold, transferred to a well of a six-well tissue culture plate, and equilibrated for minimally 2 h with growth medium. Per agarose gel, 600,000 Hepa1-6 cells, were seeded in the microwells. Cells were allowed to settle into the microwells, and the entire gel was submerged in growth medium and incubated for 40 h at 37°C and 5% CO2. Uniformly sized spheroids (~150 µm diameter) were picked from the microwells using a Pasteur pipette, mixed with 1 mg/ml collagen type I gel solution and added on top of a previously polymerized collagen matrix layer (rat tail collagen Type I (BD Biosciences), 1 mg/ml) in multiwell plates (several spheroids/well). 60 min incubation at 37°C and 5% CO2 allowed for polymerization of the spheroid-containing matrix and growth medium containing 50 ng/ml Hepatocyte growth factor (Sigma, Diegem, Belgium) and no (control) or 2 mM TUDCA was added on top. Phase contrast images of the spheroids were taken using an Olympus Cell^{M} system (magnification 50×) at time 0, 24, 48 and 60 h. Quantification of the sphere area and perimeter was obtained using ImageJ [1].

Boyden chamber invasion assay

The invasion of Hepa1-6 cells was assessed in a Boyden chamber with a 8 µm polycarbonate membrane pre-coated with basement membrane extract (BME) using the CultreCoat® 96 Well Medium BME Cell Invasion Assay (Trevigen, Gaithersburg, MD, USA) according to the manufacturer's instructions. In brief, Hepa1-6 cells were serum starved for 16 hours and seeded at 25,000 cells per well in the pre-coated chambers, which were rehydrated for one hour. Cells invaded in response to 10% FBS added to the bottom chambers over a 48 hour period and were quantitated using Calcein AM. Fluorescence was read at 485 nm excitation/520 nm emission. Samples were run in quadruplicate.

Zymography

Hepa1-6 cells were left untreated or pretreated for 12 h with 1 or 2 mM TUDCA, in the presence or absence of 50 ng/ml HGF in normal growth medium. Gelatin zymography was performed as described in [3], with some adaptations. In brief, 1×10^6 of the pretreated cells, were seeded in a 12-well plate in a 1 mg/ml collagen matrix that was allowed to polymerize for 1 h at 37°C. Subsequently 500 µl serum free medium supplemented with HGF, 1 mM or 2 mM TUDCA (as indicated) was added. After 36 h incubation, the conditioned medium was collected and concentrated 10-fold (Microcon YM-10, Millipore). Equal amounts of the concentrated media of the different conditions were analyzed on SDS-PAGE containing 0.1% gelatin (Sigma). Proteins were enabled to renature by removing SDS with 2% Triton X-100 washing buffer. Digestion occurred by overnight incubation in MMP buffer (50 mM Tris, 10 mM CaCl2, 50 mM NaCl, pH 7.5) and gels were stained using Coomassie Blue Brilliant.

REFERENCES

1. Schneider CA, Rasband WS, Eliceiri KW. NIH Image to ImageJ: 25 years of image analysis. Nat Methods. 2012; 9:671–675. doi:10.1038/nmeth.2089.
2. Napolitano AP, Dean DM, Man AJ, Youssef J, Ho DN, Rago AP, et al. Scaffold-free three-dimensional cell culture utilizing micromolded nonadhesive hydrogels. Biotechniques. 2007; 43:496–500. doi:10.2144/000112591.
3. Audenhove I, Boucherie C, Pieters L, Zwaenepoel O, Vanloo B, Martens E, et al. Stratifying fascin and cortactin function in invadopodium formation using inhibitory nano-bodies and targeted subcellular delocalization. FASEB J. 2014; 28:1805–1818. doi: 10.1096/fj.13-242537.

SUPPLEMENTARY FIGURES AND TABLES

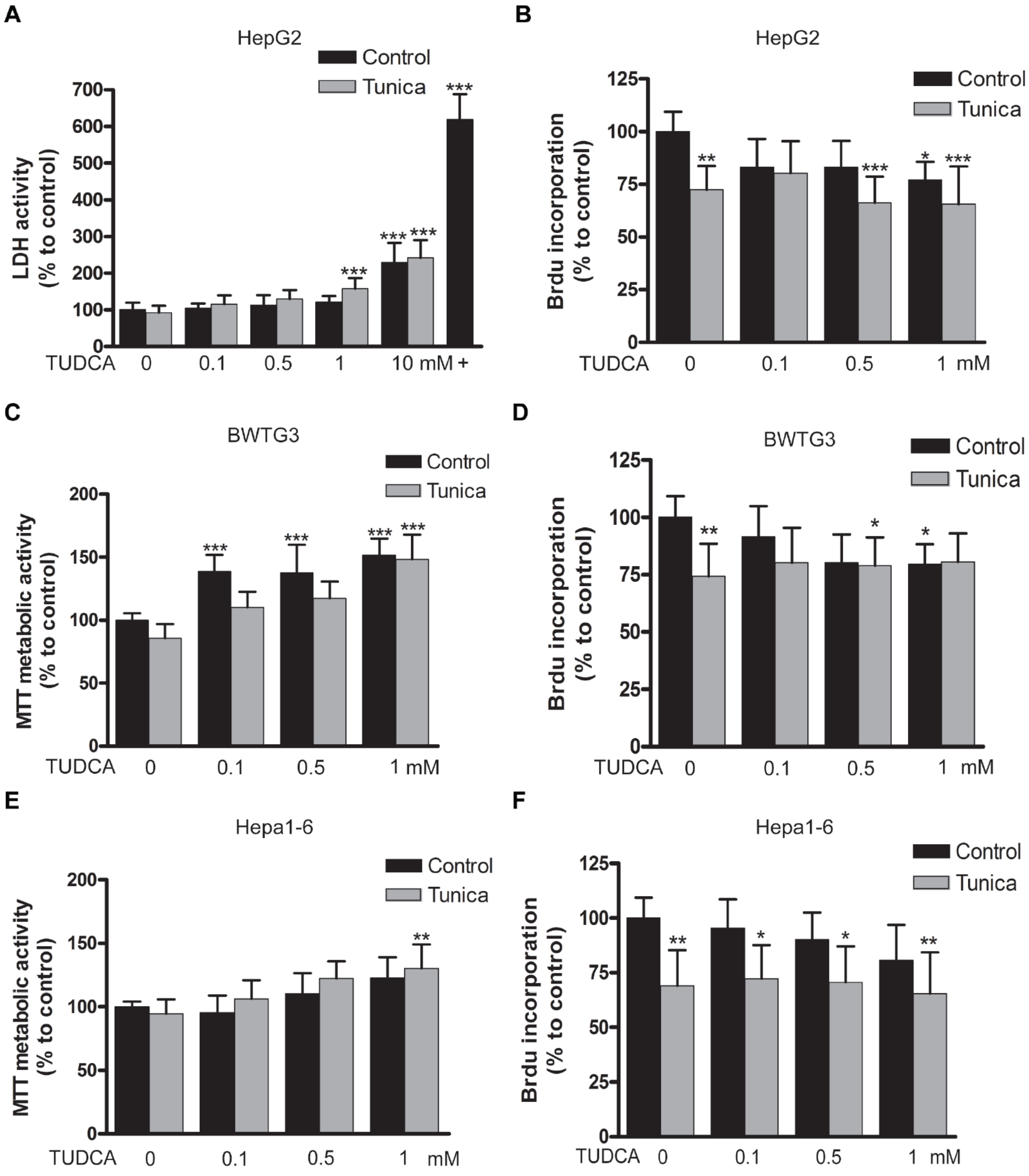

Supplementary Figure S1: Effect of TUDCA on viability and proliferation of HCC cells. Control cells or cells treated with 0.5 microgram/ml tunicamycin were treated with increasing concentrations of TUDCA as indicated. **A.** LDH release in HepG2 cells. As positive control, TritonX 1% was applied. **B.** Proliferation rate as assessed by BrdU incorporation in HepG2 cells. **C.** MTT metabolic activity and **D.** proliferation rate in BWTG3 cells. **E.** MTT metabolic activity and **F.** proliferation rate in Hepa1-6 cells. Data are presented as the mean ± SD of $n = 4$. Statistical significance was determined by one-way analysis of variance (ANOVA) with Bonferroni correction. *$p < 0.05$, **$p < 0.01$, ***$p < 0.001$.

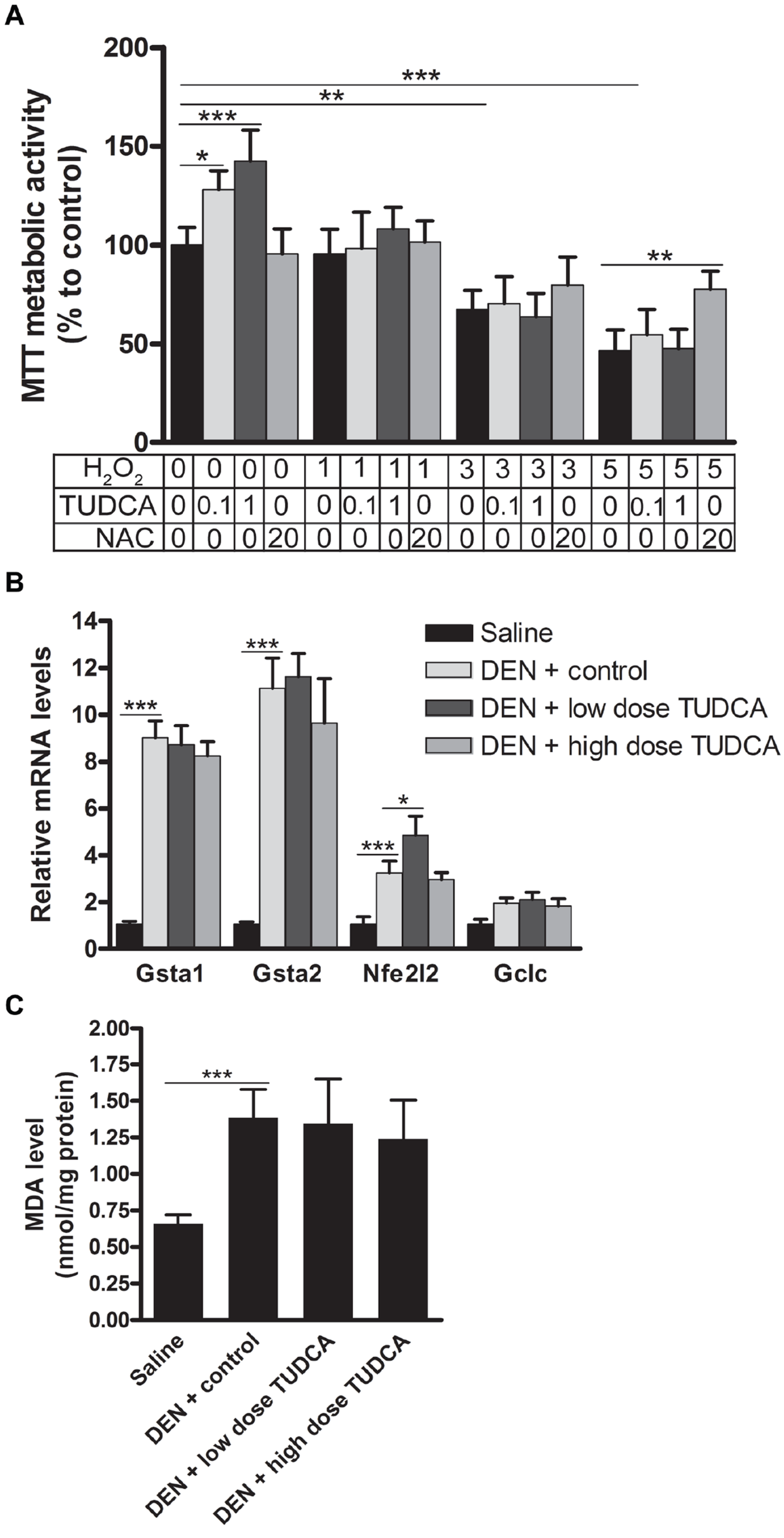

Supplementary Figure S2: Effect of TUDCA on oxidative stress-induced cytotoxicity in HepG2 cells. A. MTT assay of HepG2 cells incubated for 48 h. Concentrations are indicated in mM. NAC, N-acetylcysteine. **B.** Real-time PCR analysis of the indicated antioxidant genes after indicated cell treatments. **C.** Malondialdehyde (MDA) levels were quantified as a measure of lipid peroxidation in the mouse livers after the indicated treatments. All the values are expressed as the mean ± SD, $n = 5$. *$p < 0.05$, **$p < 0.01$, ***$p < 0.001$.

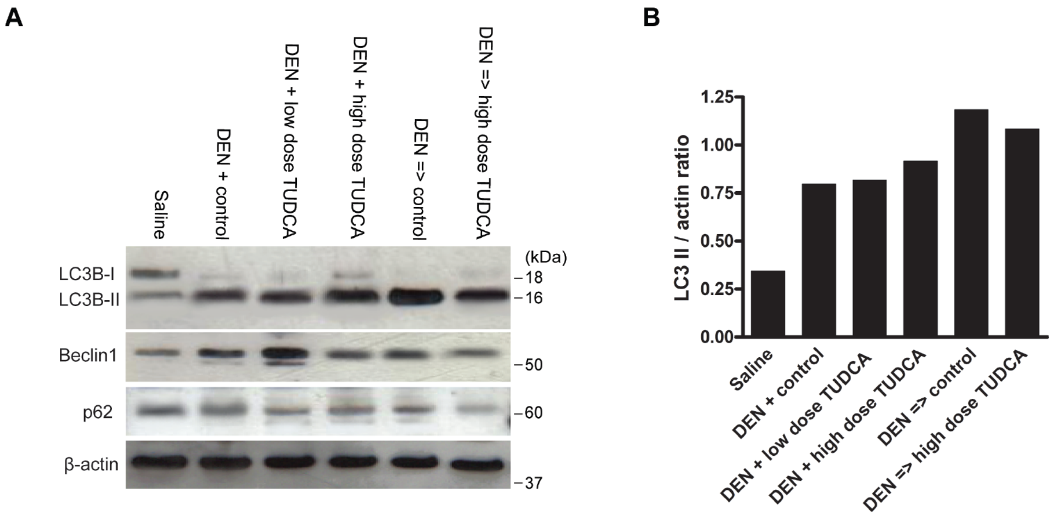

Supplementary Figure S3: Effect of TUDCA on DEN-induced autophagy. A. The expression level of autophagy-associated proteins in the livers was analyzed by western blotting, β-actin: loading control. **B.** Densitometric analysis of LC3 II/actin ratio.

Supplementary Table S1: Primers used for the qRT-PCR experiments. The PCR-efficiency of each primer pair was calculated using a standard curve of reference cDNA. Amplification efficiency R^2 was determined using the formula 10^(–1/slope).

Gene symbol	Reference sequence	Species	Forward primer	Reverse primer	Efficiency	R^2
Gapdh	NM_008084.2	*Mus musculus*	GCCGGCTCAGT GAGACAAG	TGGCACCTTCA GCAACAATG	95.1	0.99
Chop	NM_007837.3	*Mus musculus*	AGCGCAACAT GACAGTGAAG	GTGTAATTCCA GGGGGAGGT	101.2	0.99
Grp78	NM_001163434.1	*Mus musculus*	TGCCGAGCTA AATTACACATTG	CCTTGTGGAG GGATGTACAGA	107.3	0.99
Gclc	NM_010295.2	*Mus musculus*	GGGAAGAGA CCCAGCGCCAC	GCACGTCCTTG TGCCGGTCC	96.2	0.99
Gsta1	NM_008181.3	*Mus musculus*	TGATGCCAGCCT TCTGACCCCT	TGGCTGCCAGG CTGTAGGAACT	91.2	0.99
Gsta2	NM_008182.3	*Mus musculus*	GGGCAACAGGC TGACCAGGG	GGCTGGCATCAA GCTCTTCAACA	96.7	0.99
Nfe2l2	NM_010902.3	*Mus musculus*	CCATTCCCGAA TTACAGTGTCTT	AACAGCGGTAG TATCAGCCAG	95.6	0.99

Supplementary Table S2: Characteristics of the antibodies used in the study. The specificity, isotype, clone number, and catalog number of the antibodies are indicated if provided.

Antigen	Antibody isotype, clone	Company	Cat no.
eIf2α	Rabbit polyclonal IgG	Cell Signaling	9721
Phospho-eIf2α	Rabbit monoclonal IgG1, 119A11	Cell Signaling	3597
Chop	Mouse monoclonal IgG2a, L63F7	Cell Signaling	2895
Grp78	Rabbit monoclonal IgG, C50B12	Cell Signaling	3177
LC3B	Mouse monoclonal IgG1, 5F10	Nanotools	0231-100
Beclin1	Mouse monoclonal IgG1, 12B4	Nanotools	0240-100
p62	Rabbit polyclonal IgG1	Cell Signaling	5114
F4/80	Rat monoclonal IgG2b, Cl:A3-1	AbD Serotec	MCA497G
Total IκBα	Rabbit polyclonal IgG	Cell Signaling	9242
phospho-NFκB p65	Rabbit monoclonal IgG, 93H1	Cell Signaling	3033
NFκB p65	Rabbit monoclonal IgG, C22B4	Cell Signaling	4764
MMP-9	Rabbit monoclonal IgG, EP1255Y	Abcam	ab137867
MMP-14	Rabbit polyclonal IgG	Abcam	ab51074
MMP-2	Rabbit polyclonal IgG	Santa Cruz	sc-10736
β-actin	Mouse monoclonal IgG1, ACTN05	Abcam	ab3280
β-tubulin	Rabbit polyclonal IgG	Abcam	ab6046
